# Study of the Profile of Patients with STDs Attending an STD Clinic in J.A.H., Gwalior

**DOI:** 10.4103/0970-0218.43235

**Published:** 2008-10

**Authors:** Ashok Mishra, Prashant Verma, Neera Marathe, Dhiraj Srivastava

**Affiliations:** Department of Community Medicine, GR Medical College, Gwalior, Madhya Pradesh - 474 009, India

## Introduction

The rapid increase in the incidence of HIV/AIDS in the last two decades has attracted efforts by our policy makers and program makers to halt its progress. The increased risk of the transmission of HIV is know to be associated with the presence of sexually transmitted diseases (STDs) and despite the presence of the National STD Control Program in India(1) the number of people with STDs remains high.

## Materials and Methods

This hospital-based study was conducted among 77 patients attending STD clinics from June to August 2006 at a teaching hospital of G.R. Medical College, Gwalior (MP) after receiving their verbal informed consent. The male and female patients were interviewed by a male and female interviewer, respectively employing a pretest and structured proforma. The study variables were analyzed using SPSS-10 software.

## Results

Out of 90 patients, 77 agreed to participate in the study. There were 42 females (54.54%) and 35 males (45.45%). The majority of patients were Hindu by religion (87.1%), followed by Muslims (10.39%). All the female patients were married while only 71.4% of the male patients were married. A majority of male attendees were educated up to the graduate level or above while amongst the females this proportion was only 12%. Three-fourths of the patients were from an urban setting. Two-thirds of the female patients were housewives while a similar proportion of the males were involved in the service sector, factories, hotel staff, and auto or taxi drivers.

All of the women were involved in heterosexual relationships; 2 out of 42 had extramarital sexual relationships with a friend (2.3%) or relative (2.3%). Out of 35 males, 3 had either homosexual or bisexual relationships; 26 (74.3%) of the male subjects reported having sex with non regular sex partners such as a friend (60%) or a commercial sex worker (31.4%); only 10 out of 26 (38%) used condoms while having sex with non regular sex partners in their last sexual encounter. About 54% and 59.6% of the men and women, respectively, reported the use of condoms while having sex with their spouses.

Using a syndromic approach among these attendees, discharge was reported as the most common complaint (100%) among female attendees followed by lower abdominal pain (61.3%), ulcers (16.6%), and nodules (11.4%) in the genitals. The nature of vaginal discharge among females was curd-like (21%), pus-like (38%), or foul smelling (41%). A large number of these attendees reported the occurrence of a similar complaint in the past. The male attendees presented with the most common complaint of ulcers in genitalia (80%) followed by discharge (14.7%), lower abdominal pain (14.7%), and nodules (11.4%) and a few had a history of similar problems in the past.

Simultaneous STD infections in spouses were observed in 4.9% of the patients and mainly comprised of ulcers (85.7%). The preferred place for treatment of STDs among these attendees was government health facilities (49.4%) followed by private practitioners (25.9%). An alarming finding was that 19 out of 77 (25%) attendees underwent self-medication or sought treatment from illegitimate sources.

## Discussion

This study findings may have implications on designing any intervention on this population. The majority (70%) of the attendees were in the age group of 25–44 years old, similar to that reported by Bhatnager, *et al.*(2) though a good number of them (17 out of 77) were younger than 25 years old. Other researchers such Ram, *et al.*(3) have reported that a significant proportion of reproductive tract infections (RTIs) were reported among adolescents. These findings suggest that intervention should start from a younger age.

Our finding of high-risk sexual behavior among adolescent males and a subsequent higher risk of STDs is similar to that reported by Subramanian, *et al.*(4) The majority of the respondents were involved in an occupation that has been traditionally considered high-risk by National AIDS Control Organization. Out of the male attendees, 28.5% were unmarried. Unmarried males with specific high-risk occupations (truck drivers, hotel staff, and factory workers) are at the highest risk of developing STDs due to their sexual promiscuity. The study also found that 71% of the attendees were from an urban background, probably due to the location of the hospital in urban area. It is a sign of concern that 74% of the STD clinic attendees were having sex with non regular sex partners and only 38.46% of them used a condom during their last sexual encounter. These findings suggest that such attendees should be given counseling at the clinic about safe sexual practices.

The reports of vaginal discharge as the most common complaint among women is also similar to the report by Kore, *et al.*(5) The most common complaint in males was ulcer (80%), whereas females presented with multiple complaints. This may be because males seek early treatment while females waited for a longer period until they developed other complaints. It is still a matter of concern that almost 25% of the attendees first relied on self-medication or went to illegitimate sources before coming to government facilities. What is more alarming is the fact that only 9 out of 77 attendees were counseled about STDs or HIV/AIDS. This reinforces the need for early referral and appropriate counseling.

## Conclusions

This study points to the need for addressing issues such as unsafe sexual practices among adolescents and the need for early referral treatment and counseling.
